# High-sensitivity troponin I and B-type natriuretic peptide biomarkers for prediction of cardiovascular events in patients with coronary artery disease with and without diabetes mellitus

**DOI:** 10.1186/s12933-019-0974-2

**Published:** 2019-12-17

**Authors:** Yuen-Kwun Wong, Chloe Y. Y. Cheung, Clara S. Tang, JoJo S. H. Hai, Chi-Ho Lee, Kui-Kai Lau, Ka-Wing Au, Bernard M. Y. Cheung, Pak-Chung Sham, Aimin Xu, Karen S. L. Lam, Hung-Fat Tse

**Affiliations:** 1 Department of Medicine, The University of Hong Kong, Queen Mary Hospital, Hong Kong, China; 2 Department of Surgery, The University of Hong Kong, Hong Kong, China; 3 Department of Psychiatry, The University of Hong Kong, Hong Kong, China; 4 Centre for Genomic Sciences, Li Ka Shing Faculty of Medicine, The University of Hong Kong, Hong Kong, China; 5 State Key Laboratory in Brain and Cognitive Sciences, The University of Hong Kong, Hong Kong, China; 6 State Key Laboratory of Pharmaceutical Biotechnology, The University of Hong Kong, Hong Kong, China; 7 Department of Pharmacology & Pharmacy, The University of Hong Kong, Hong Kong, China; 8 Department of Medicine, Shenzhen Hong Kong University Hospital, Shenzhen, China; 9 Hong Kong-Guangdong Joint Laboratory on Stem Cell and Regenerative Medicine, The University of Hong Kong, Hong Kong, China; 10 Shenzhen Institutes of Research and Innovation, The University of Hong Kong, Hong Kong, China

**Keywords:** B-type natriuretic peptide, Cardiac troponin, Coronary artery disease, Risk prediction, Type 2 diabetes mellitus

## Abstract

**Background:**

High-sensitivity troponin I (hs-Tnl) and B-type natriuretic peptide (BNP) are promising prognostic markers for coronary artery disease (CAD). This prospective cohort study investigated whether a combination of these cardiac biomarkers with conventional risk factors would add incremental value for the prediction of secondary major adverse cardiovascular events (MACEs) in patients with CAD, with and without type 2 diabetes mellitus (T2DM).

**Methods:**

Baseline plasma level of hs-Tnl and BNP was measured in 2275 Chinese patients with stable CAD. Patients were monitored for new-onset of MACE over a median of 51 months. Cox proportional hazard model and area under the receiver operating characteristic curve (AUC) were used to assess the association of cardiac biomarkers with MACE and their predictive values in relationship with or without T2DM.

**Results:**

During the follow up period 402 (18%) patients experienced a new-onset MACE with hs-Tnl and BNP level significantly higher than in those without MACE. In multivariable analyses, patients with elevated hs-Tnl (hazard ratio, 1.75 [95% CI 1.41–2.17]; *P *< 0.001) and BNP (hazard ratio, 1.42 [95% CI 1.15–1.75]; *P *= 0.001) were significantly associated with an increased risk of MACE after adjustment for variables of a risk factor model of age, sex, T2DM and hypertension. The risk factor model had an AUC of 0.64 for MACE prediction. The AUC significantly increased to 0.68 by the addition of hs-Tnl to the risk factor model. Subgroup analyses showed that hs-Tnl and BNP remained significant predictors of MACE in both patients with and without T2DM in multivariable models with higher risk of MACE evident in those without T2DM. Among patients without T2DM, addition of each biomarker yielded greater predictive accuracy than in T2DM patients, with AUC further increased to 0.75 when a combination of hs-Tnl and BNP was added to the risk factor model (age, sex and hypertension).

**Conclusions:**

Elevated hs-Tnl and BNP level are independent predictors of new-onset MACE in CAD patients, irrespective of diabetes status. Among CAD patients without T2DM, a combination of cardiac biomarkers hs-Tnl and BNP yield the greatest predictive value beyond conventional risk factors.

## Background

Conventional risk factors that include age, gender, smoking, glucose level, blood pressure and cholesterol level have historically been used to risk stratify subjects who are at risk of major adverse cardiovascular events (MACEs) [1, 2]. Nevertheless these clinical risk factors on their own have limited predictive value in patients with established coronary artery disease (CAD) and in whom more frequent surveillance and aggressive risk factor control is desired. Recently, cardiac biomarkers have been shown to be important in prediction of cardiovascular risk and superior to models based on only conventional risk factors [3, 4]. High-sensitivity troponin I (hs-Tnl), a well-known diagnostic marker of myocardial injury, has been shown to be associated with the burden of coronary atherosclerosis and impaired cardiac performance [5, 6]. Elevated hs-Tnl concentration has been demonstrated to be associated with cardiovascular comorbidities and a strong predictor of adverse cardiovascular outcomes, particularly in patients with stable CAD or type 2 diabetes mellitus (T2DM), and in the elderly population [7–11]. B‐type natriuretic peptide (BNP), a widely used marker for early diagnosis of acute heart failure and for risk stratification of patients with congestive heart failure [12, 13], has also been shown to be highly predictive of the occurrence of acute coronary syndromes (ACS) and mortality [14–17]. It is unknown whether a combination of hs-TnI and BNP can provide any incremental benefit for risk prediction in patients with established CAD. In addition, T2DM is a known predictor of elevated hs-Tnl and BNP [18, 19], and in patients with heart failure, the presence of diabetes is associated with a higher BNP level [20]. It remains unclear whether these cardiac biomarkers provide different predictive abilities for subsequent cardiovascular events in CAD patients with and without T2DM.

In this study, we sought to investigate the association of these cardiac biomarkers with long-term adverse cardiovascular outcomes and to determine whether adoption of a multiple cardiac biomarkers approach can provide incremental benefit beyond a conventional risk factor approach in patients with stable CAD, as well as their predictive value in subgroups of CAD patients with or without T2DM.

## Methods

### Study population

In this prospective cohort study, we recruited consecutive patients with stable CAD who attended follow-up at the Cardiac Clinic, Queen Mary Hospital, Hong Kong from December 2003 to December 2014. All study participants received evidence-based medical therapies including coronary revascularization and statins. Diagnosis of stable CAD was defined according to the guidelines of the American College of Cardiology [21]. The study was approved by the local Institutional Review Board and all patients provided written informed consent.

### Baseline and laboratory measurements

Comprehensive data on demographics, medical history, medication use, smoking status and anthropometric parameters including body mass index and blood pressure were recorded at enrollment. Missing anthropometric measurements were imputed using multiple imputation in R package MICE [22]. Patients with any tobacco use in the last 30 days were considered a current smoker. T2DM was defined according to the World Health Organization 1998 diagnostic criteria [23], or regular prescription of anti-diabetic medication. Hypertension was considered present in patients with a history of hypertension or regularly prescribed anti-hypertensive medication.

Blood samples were drawn at recruitment from all patients following an overnight fast of 12 h to measure lipid profiles, glucose, hs-Tnl and BNP. Samples were stored at − 70 °C until analysis. Plasma hs-Tnl was measured by ARCHITECT STAT high-sensitive troponin-I assay (Abbott Laboratories, Abbott Park, IL, USA). The limit of detection was 1.9 pg/mL and the coefficient of variation at 99th percentile was 4%. Plasma BNP level was measured using ARCHITECT BNP assay (Abbott Laboratories, Abbott Park, IL, USA), measurement range 10–5000 pg/mL.

### Outcomes

Follow-up for each patient began at enrollment and continued until diagnosis of a cardiovascular end-point, death, last visit, or end of study follow-up, whichever came first. Cardiovascular end-point was new-onset MACE during the follow-up period. Diagnosis of MACE was based on the International Classification of Disease Ninth Revision (ICD-9), and included acute myocardial infarction (ICD-9 410), ACS (ICD-9 411.1), stroke (ICD-9 430, 431, 433, 434, 436), peripheral vascular disease (ICD-9 443.9), and cardiovascular death (death certificate ICD-9 410-447). Follow-up information including dates of events and discharge diagnosis were verified from medical records of the Hong Kong Hospital Authority database. The main cause and date of death were obtained from the Hong Kong Death Registry for patients who died during the study period.

### Statistical analysis

Data are presented as mean ± standard deviation or number and percentage, as appropriate. Kolmogorov–Smirnov test was used to determine the normality assumption for continuous variables. We transformed variables with a skewed distribution using natural logarithm transformation before analysis. Age was categorized as younger than 65 years or 65 years and over based on our recent study [4]. The optimal cutoff value for each biomarker was determined by Youden J index. Comparisons between groups were evaluated using Student’s t test for continuous variables and Chi squared test for categorical variables.

Survival curves were estimated using Kaplan–Meier method and the cumulative incidence of MACE for each biomarker was compared using log-rank test. Cox proportional hazard analyses were performed to examine the association between biomarker levels and MACE, adjusting for age, clinical risk factors including sex, current smoker, T2DM, hypertension and body mass index, as well as enrollment period, which was considered a confounder influencing prognosis. As opposed to baseline blood pressure and hemoglobin A1c measurements, history of T2DM and hypertension were used in the model selection to reduce variations caused by factors such as anti-hypertensive or anti-diabetic medication use. The proportional hazards assumption in each Cox regression model was tested using Schoenfeld residuals and no violation was observed. Tests for the interactions of age, sex and T2DM status with cardiac biomarkers were also performed. The independent association of biomarker level with MACE was evaluated in various models. Model 1: age ≥ 65 years. Model 2: age ≥ 65 years, clinical risk factors and enrollment period; variables were chosen if they were associated with MACE in the age-adjusted models with *P *< 0.10 to avoid over-adjustment. Model 3 to 5: additionally included cardiac biomarkers alone or in combination with the clinical risk factor model. Sensitivity analysis was conducted by using the competing risks regression model of Fine and Gray to estimate the sub-hazard ratios for MACE, with non-cardiovascular deaths treated as competing risk [24].

The area under the receiver operating characteristic curve (AUC), category-free net reclassification index and integrated discrimination improvement were used to assess the incremental predictive value of a model. The differences between AUCs were compared using DeLong’s test [25]. The performance of Cox regression models was evaluated by using the Akaike Information Criterion for model comparisons, where a lower Akaike Information Criterion value indicates a better fit. Based on the findings from a previous study [26], assuming the prediction model with cardiac biomarkers and clinical risk factor to detect a hazard ratio of 1.50 for cardiovascular outcomes, 198 events were required to provide an 80% power with a 2-sided significance level of 0.05. Effective sample size was achieved based on the rule of ten outcome events per predictor to develop the risk prediction model using Cox proportional hazards regression [27].

A two-sided *P* value less than 0.05 was considered statistically significant. All statistical tests were performed using STATA statistical software (version 14.0) and R-programming language (version 3.5.1).

## Results

### Baseline characteristics and biomarker levels

The demographic and biochemical characteristics with respect to incident MACE during follow-up are summarized in Table 1. Patients with missing clinical or biomarker data (n = 177) were excluded and 2275 patients (mean age: 67.7 ± 10.5 years) were included in the study. Among all patients with stable CAD, 402 experienced new-onset MACE after a median follow-up of 51 months (incidence rate, 4.2 per 100 patient-years). Based on the Youden J index, the optimal cutoff plasma level of hs-Tnl and BNP for prediction of MACE was 14.2 pg/mL and 56.7 pg/mL, respectively. Affected CAD patients were significantly older, and a higher proportion were female, and had T2DM or hypertension compared with those with no MACE during follow-up (all *P *< 0.01). Patients with CAD and incident MACE also had higher levels of plasma hs-Tnl and BNP, hemoglobin A1c and systolic blood pressure, but lower diastolic blood pressure at baseline (all *P *< 0.001). Nonetheless no differences in body mass index or cholesterol level were evident between CAD patients with or without incident MACE (*P *> 0.05).

**Table 1:** Baseline characteristics of the study cohort

Variable	All	Subjects with major adverse cardiovascular events	Subjects without major adverse cardiovascular events	*P* value
N	2275	402	1873	
Age ≥ 65 years	1400 (62)	313 (78)	1087 (58)	< 0.001
Male	1664 (73)	270 (67)	1394 (74)	0.003
Current smoker	289 (13)	49 (12)	240 (13)	0.73
T2DM	1617 (71)	328 (82)	1289 (69)	< 0.001
Hypertension	1997 (88)	380 (95)	1617 (86)	< 0.001
BMI, kg/m^2^	25.8 ± 3.6	25.4 ± 3.9	25.8 ± 3.6	0.054
Systolic blood pressure, mmHg	137 ± 20	140 ± 23	137 ± 20	< 0.001
Diastolic blood pressure, mmHg	73 ± 11	70 ± 12	74 ± 11	< 0.001
Triglycerides, mmol/L	1.49 ± 1.02	1.51 ± 1.11	1.49 ± 1.00	0.65
Total cholesterol, mmol/L	3.89 ± 0.89	3.94 ± 1.01	3.88 ± 0.87	0.17
HDL-cholesterol, mmol/L	1.18 ± 0.33	1.18 ± 0.33	1.18 ± 0.33	0.79
LDL-cholesterol, mmol/L	2.01 ± 0.73	2.06 ± 0.83	2.01 ± 0.71	0.22
HbA1c, %	7.07 ± 1.39	7.41 ± 1.51	7.00 ± 1.36	< 0.001
Ln-hs-Tnl, pg/mL	2.1 ± 1.2	2.5 ± 1.4	2.1 ± 1.1	< 0.001
Above cutoff (> 14.2 pg/mL)	511 (22)	148 (37)	363 (19)	< 0.001
Ln-BNP, pg/mL	3.8 ± 1.1	4.1 ± 1.3	3.7 ± 1.1	< 0.001
Above cutoff (> 56.7 pg/mL)	877 (39)	212 (53)	665 (36)	< 0.001
Ln-Enrollment period, month	3.1 ± 1.0	3.5 ± 0.9	3.0 ± 1.0	< 0.001

Values are mean ± SD or n (%)

*BMI* body mass index, *BNP* B-type natriuretic peptide, *HbA1c* hemoglobin A1c, *HDL-C* high density lipoprotein cholesterol, *hs-Tnl* high-sensitivity troponin I, *Ln* natural logarithm, *LDL-C* low density lipoprotein cholesterol, *T2DM* type 2 diabetes mellitus

Baseline characteristics of study participants according to T2DM status are presented in Table 2. There were 1617 (71%) CAD patients had T2DM. They were older and more often had a history of hypertension than those without T2DM. Patients with T2DM also had a higher BNP concentration and body mass index, lower total cholesterol and low-density lipoprotein and similar hs-Tnl concentration compared with patients without T2DM. The annual event rate of MACE in T2DM CAD patients (4.6 per 100 patient-years) was significantly higher than for those without T2DM (3.0 per 100 patient-years; *P *< 0.001).

**Table 2:** Comparison of baseline characteristics between patients with and without T2DM

Variables	T2DM	No T2DM	*P* value
n	1617	658	
Age ≥ 65 years	1029 (64)	371 (56)	0.001
Male	1168 (72)	496 (75)	0.13
Current smoker	181 (11)	108 (16)	0.001
Hypertension	1557 (96)	440 (67)	< 0.001
BMI, kg/m^2^	26.0 ± 3.6	25.2 ± 3.6	< 0.001
Systolic blood pressure, mmHg	140 ± 21	131 ± 18	< 0.001
Diastolic blood pressure, mmHg	73 ± 11	74 ± 11	0.066
Triglycerides, mmol/L	1.50 ± 1.02	1.46 ± 1.02	0.37
Total cholesterol, mmol/L	3.80 ± 0.85	4.09 ± 0.97	< 0.001
HDL-cholesterol, mmol/L	1.16 ± 0.33	1.23 ± 0.34	< 0.001
LDL-cholesterol, mmol/L	1.95 ± 0.67	2.17 ± 0.84	< 0.001
HbA1c, %	7.38 ± 1.36	6.34 ± 1.17	< 0.001
Ln-hs-Tnl, pg/mL	2.2 ± 1.1	2.1 ± 1.4	0.44
Above cut-off (> 14.2 pg/mL)	376 (23)	135 (21)	0.16
Ln-BNP, pg/mL	3.8 ± 1.1	3.6 ± 1.2	< 0.001
Above cut-off (> 56.7 pg/mL)	672 (42)	205 (31)	<0.001
Ln-enrollment period, month	3.1 ± 0.8	2.9 ± 1.3	<0.001
Outcomes			
Major adverse cardiovascular events	328 (20)	74 (11)	< 0.001

Values are mean ± SD or n (%)

*BMI* body mass index, *BNP* B-type natriuretic peptide, *HbA1c* hemoglobin A1c, *HDL-C* high density lipoprotein cholesterol, *hs-Tnl* high-sensitivity troponin I, *Ln* natural logarithm, *LDL-C* low density lipoprotein cholesterol, *T2DM* type 2 diabetes mellitus

### Predictive values of cardiac biomarkers for MACE

**Fig. 1:**
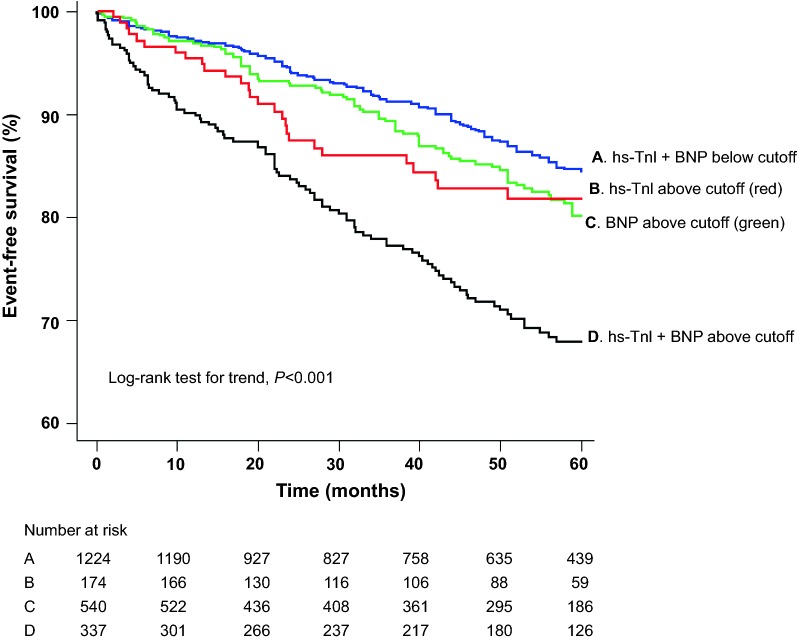
Kaplan-Meier survival curves for MACEs in stable CAD patients. *BNP* B-type natriuretic peptide, *hs-Tnl* high-sensitivity troponin I

As shown in Fig. 1, Kaplan–Meier event-free curves for MACE show that patients with plasma hs-Tnl and BNP level above optimal cutoff value had significantly worse survival than those with levels below (log-rank test for trend *P *< 0.001). In the age-adjusted Cox regression analysis, sex, T2DM and hypertension were associated (*P *< 0.10) with an increased risk of MACE and were included in the final model (Additional file 1: Table S1). In the multivariable Cox proportional hazards model, higher level of individual biomarkers was strongly associated with an increased risk of subsequent MACE in all patients with stable CAD, after adjustment for advanced age, sex, T2DM and hypertension. The adjusted hazard ratio for patients with elevated level of hs-Tnl or BNP was 1.75 (95% CI 1.41–2.17; *P *< 0.001) and 1.42 (95% CI 1.15–1.75; *P *= 0.001), respectively (Table 3; Model 5).

**Table 3:** Multivariable Cox regression models predicting MACEs in stable CAD patients

Variable	Model 1		Model 2		Model 3		Model 4		Model 5	
	HR (95% CI)	*P* value	HR (95% CI)	*P* value	HR (95% CI)	*P* value	HR (95% CI)	*P* value	HR (95% CI)	*P* value
Age ≥ 65 years	2.53 (2.00–3.21)	< 0.001	2.36 (1.85–2.99)	< 0.001	2.29 (1.80–2.91)	< 0.001	2.31 (1.82–2.93)	< 0.001	2.28 (1.79–2.90)	< 0.001
Male			0.83 (0.68–1.03)	0.089	0.84 (0.68–1.04)	0.098	0.85 (0.69–1.05)	0.14	0.85 (0.69–1.06)	0.15
T2DM	–	–	1.25 (0.95–1.65)	0.11	1.24 (0.94–1.63)	0.13	1.30 (0.99–1.71)	0.062	1.28 (0.97–1.69)	0.079
Hypertension	–	–	1.58 (1.00–2.50)	0.051	1.54 (0.97–2.45)	0.065	1.57 (0.99–2.49)	0.055	1.55 (0.98–2.46)	0.063
BNP	–	–	–	–	1.66 (1.36–2.02)	<0.001	–	–	1.42 (1.15–1.75)	0.001
hs-Tnl	–	–	–	–	–	–	1.97 (1.60–2.41)	<0.001	1.75 (1.41–2.17)	< 0.001

BNP and hs-Tnl are at levels above optimal cutoffs

*BNP* B-type natriuretic peptide, *CAD* coronary artery disease, *CI* confidence interval, *HR* hazard ratio, *hs-Tnl* high-sensitivity troponin I, *MACE* major adverse cardiovascular event, *T2DM* type 2 diabetes mellitus

After accounting for the competing risk of non-cardiovascular death, the association of cardiac biomarkers with incident MACE remained significant and largely unchanged (Additional file 1: Table S2).

### Incremental value of cardiac biomarkers over conventional risk factors for MACE

**Fig. 2:**
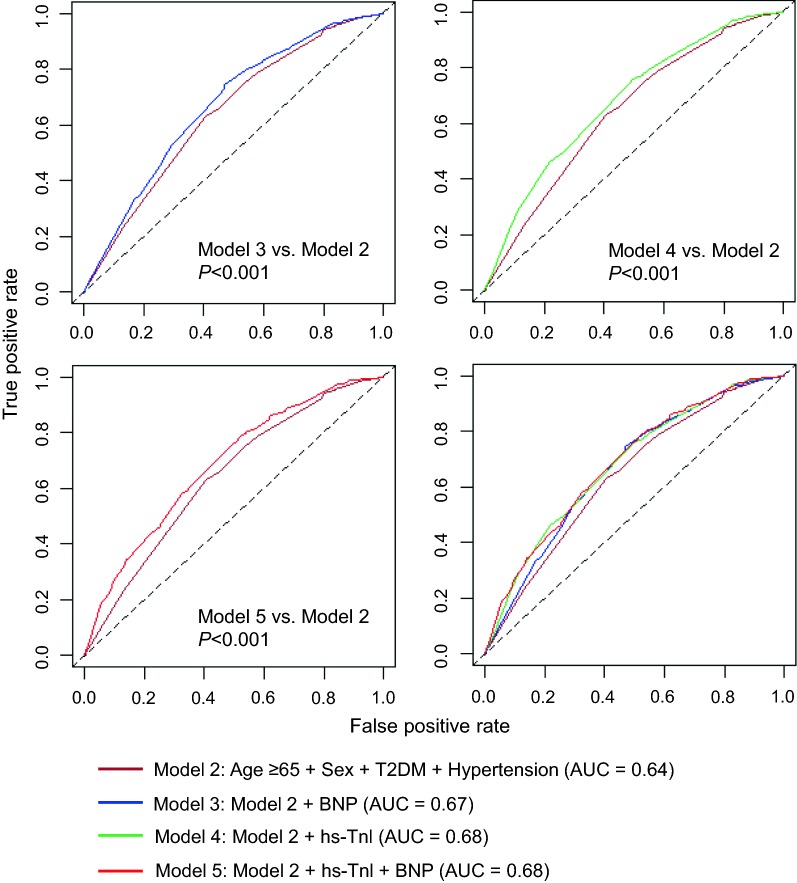
Area under the receiver operating characteristic curves for predicting MACEs in stable CAD patients. *BNP* B-type natriuretic peptide, *hs-Tnl* high-sensitivity troponin I, *T2DM* type 2 diabetes mellitus

The receiver operating characteristic curve for different models are shown in Fig. 2. The AUC was 0.60 (95% CI 0.58–0.62) for model with age ≥ 65 years alone (Model 1). The addition of sex, T2DM and hypertension to the age model significantly increased the AUC to 0.64 (95% CI 0.61–0.67; Model 2). Moreover, significant increase in AUCs were observed when each biomarker was individually added to the risk factor model: hs-Tnl had the best single biomarker model with AUC further increased to 0.68 (95% CI 0.65–0.71; difference in AUCs, 0.04; *P *< 0.001; Model 4), whereas BNP yielded a slightly lower value of 0.67 (95% CI 0.64–0.69; difference in AUCs, 0.03; *P *= 0.001; Model 3) (Fig. 2). As shown in Table 4, both single biomarker models significantly improved risk reclassification and discrimination (hs-Tnl: net reclassification index [NRI], 34.9%; 95% CI 24.8–45.0; integrated discrimination improvement [IDI], 2.4%; 95% CI 1.6–3.2; BNP: NRI, 34.5%; 95% CI 23.8–45.1; IDI, 1.2%; 95% CI 0.7–1.7). According to the Akaike Information Criterion, the addition of a single or combination of biomarkers to the risk factor model improved model prediction for MACE (Table 4). In contrast, incorporating a combination of hs-Tnl and BNP into the age and clinical risk factor model (Model 5: AUC, 0.68; 95% CI 0.65–0.71) did not result in an increase in AUC when compared with the single biomarker model (Model 5 versus Model 4: DeLong’s test, *P *= 0.55).

**Table 4:** Performance of models predicting MACEs in stable CAD patients

Model	Feature	AIC	NRI (95% CI)	*P* value	IDI (95% CI)	*P* value
Model 2	Age ≥ 65 years + Male + T2DM + Hypertension	5701.9	–	–	–	–
Model 3	Model 2 + BNP	5678.3	34.5% (23.8–45.1%)	< 0.001	1.2% (0.7–1.7%)	< 0.001
Model 4	Model 2 + hs-Tnl	5664.5	34.9% (24.8–45.0%)	< 0.001	2.4% (1.6–3.2%)	< 0.001
Model 5	Model 2 + hs-Tnl + BNP	5655.5	29.7% (19.2–40.2%)	< 0.001	2.9% (2.1–3.8%)	< 0.001

Model 2: incorporated variables of age ≥ 65 years, male sex, presence of T2DM and hypertension; Model 3: variables in Model 2 and addition of BNP above optimal cutoff; Model 4: variables in Model 2 and addition of hs-Tnl above optimal cutoff; Model 5: variables in Model 2 and addition of hs-Tnl and BNP above cutoffs

*AIC* Akaike Information Criteria, *BNP* B-type natriuretic peptide, *CAD* coronary artery disease, *CI* confidence interval, *hs-Tnl* high-sensitivity troponin I, *IDI* integrated discrimination improvement, *MACE* major adverse cardiovascular event, *NRI* net reclassification index, *T2DM* type 2 diabetes mellitus

Interaction analyses showed significant interactions of T2DM status with hs-Tnl (*P*_interaction_ = 0.046), and BNP (*P*_interaction_ = 0.010), whereas no interactions of age and sex with biomarkers (all *P*_interaction_ > 0.05) on MACE were found in the multivariable models.

### Cardiac biomarkers for patients with or without T2DM

In multivariable Cox regression analyses, baseline hs-Tnl and BNP level remained significant predictors of MACE in both patients with and without T2DM after adjustment for age, sex and hypertension with higher risk of MACE evident in those without T2DM (Additional file 1: Table S3).

**Fig. 3:**
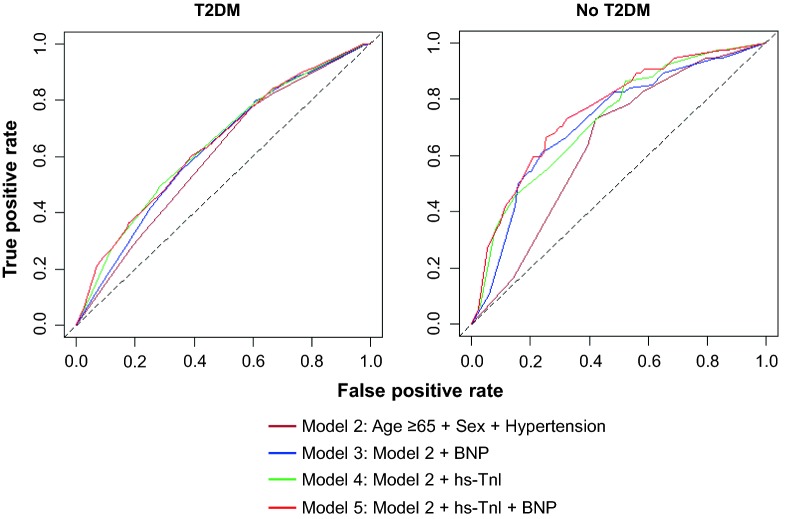
Area under the receiver operating characteristic curves for predicting MACEs according to T2DM status. *BNP* B-type natriuretic peptide, *hs-Tnl* high-sensitivity troponin I, *T2DM* type 2 diabetes mellitus

The predictive value of different models for MACE according to T2DM status are presented in Table 5 and Fig. 3. In patients with or without T2DM, the addition of any biomarker to the age and clinical risk factor model showed a significant increase in AUC for the prediction of MACE. Among patients with T2DM, the AUCs increased from 0.60 (95% CI 0.57–0.63) to 0.63 (95% CI 0.59–0.66) and 0.64 (95% CI 0.61–0.67) for BNP and hs-Tnl (DeLong’s test, both *P *< 0.001), respectively. Interestingly, the predictive performance of single biomarker models in patients without T2DM were significantly better than for those with T2DM (T2DM versus no T2DM, Model 3–5, all *P *< 0.05), with AUC increased from 0.64 (95% CI 0.59–0.70) to 0.72 (95% CI 0.66–0.78; DeLong’s test, both *P *< 0.01).

**Table 5:** Subgroup analysis for performance of models predicting MACEs in patients with and without T2DM

Model	Feature	T2DM, AUC (95% CI)	No T2DM, AUC (95% CI)	*P* value*
Model 2	Age ≥ 65 years + Male + hypertension	0.60 (0.57–0.63)	0.64 (0.59–0.70)	0.23
Model 3	Model 2 + BNP	0.63 (0.59–0.66)	0.72 (0.66–0.78)	0.009
Model 4	Model 2 + hs-Tnl	0.64 (0.61–0.67)	0.72 (0.66–0.78)	0.016
Model 5	Model 2 + hs-Tnl + BNP	0.64 (0.61–0.68)	0.75 (0.69–0.81)	<0.001

BNP and hs-Tnl are at levels above optimal cutoffs

*AUC* area under the curve, *BNP* B-type natriuretic peptide, *CI* confidence interval, *hs-Tnl* high-sensitivity troponin-I, *T2DM* type 2 diabetes mellitus

*P value from DeLong’s test for difference between AUCs of T2DM versus no T2DM

Adding a combination of hs-Tnl and BNP to the risk factor model further increased the AUC to 0.75 (95% CI 0.69–0.81), incremental benefit was observed in patients without T2DM when compared with the single biomarker model (Model 5 versus Model 4: DeLong’s test, *P *= 0.047). In contrast, among patients with T2DM, the combination of two cardiac biomarkers offered no significant incremental value over the single biomarker model in the risk prediction of MACE (Model 5 versus Model 4: DeLong’s test, *P *= 0.45).

## Discussion

### Main findings

In this cohort study of 2275 patients with stable CAD, higher baseline levels of hs-Tnl and BNP were strongly associated with subsequent risk of MACE. Baseline hs-Tnl and BNP level were independent predictors of incident MACE in both patients with and without T2DM, and remained significant after adjustment for age and clinical risk factors. The addition of each biomarker to the age and clinical risk factor model offered individual incremental benefit over conventional risk factors for predicting MACE. Among patients without T2DM, these two cardiac biomarkers, alone and in combination, provided greater improvements in risk prediction than in those with T2DM. It is of note that the incremental benefit of a combination of the two cardiac biomarkers over individual cardiac biomarker model of hs-Tnl was observed only in patients without T2DM. Our findings confirm the incremental prognostic value of hs-Tnl and BNP beyond conventional risk factors, and the superior predictive performance of the combined cardiac biomarkers models in patients with stable CAD without T2DM.

### Predictive values of cardiac biomarkers for MACE

In this study, we examined the prognostic value of two cardiac biomarkers that reflect different pathophysiological mechanisms, including myocardial injury (hs-Tnl) and wall stress (BNP) for prediction of MACE. Prior studies have shown that hs-Tnl is a highly specific marker for cardiac damage and is associated with increased risk of cardiovascular events and mortality in the elderly and patients with chest pain [7, 9, 28, 29]. Our recent study also demonstrated that elevated hs-Tnl was associated with increased MACE in CAD patients with statin intolerance [30]. Moreover, BNP has been shown to be an important prognostic marker of left ventricular dysfunction as well as a predictor of cardiovascular morbidity and mortality in patients with stable vascular disease [31, 32]. Previous studies have demonstrated that plasma BNP level is increased in the presence of heart failure, myocardial infarction, left ventricular hypertrophy and diabetes [33–35]. In patients with prior ACS, recent studies [36] show that elevated levels of cardiac troponin and BNP are independently associated with higher risk of recurrent cardiovascular events. Similarly, these biomarkers have shown to be an independent prognostic marker for cardiovascular events and all-cause mortality in patients with stable CAD [37, 38].

In this study, we confirmed these findings and demonstrated that elevated hs-Tnl and BNP level was associated with increased risk of MACE in CAD patients with or without T2DM. The annual incidence rate of MACE was 8.0% among patients with elevated levels of hs-Tnl and BNP at baseline, compared with 3.0% among those without elevated cardiac biomarkers, supporting the utility of hs-Tnl and BNP as prognostic markers for future cardiovascular events in patients with stable CAD. Moreover, we performed a direct comparison of cardiac biomarkers for risk prediction of MACE in these patients. Among them, elevated hs-Tnl level provided a higher AUC and was associated with 1.9-fold increased risk of MACE, whereas a slightly lower AUC was observed for elevated BNP with an association of 1.6-fold increased risk.

### Predictive values of cardiac biomarkers in CAD patient with or without T2DM

It is well known that T2DM is a predictor of elevated hs-Tnl [18, 19]. On the other hand, an elevated hs-TnI level was associated with the occurrence of MACE in patients with T2DM [30, 39]. Similarly, elevated BNP was associated with adverse cardiovascular outcomes in patients with T2DM [40]. Moreover, in T2DM patients with recent ACS, elevated BNP level was predictive of MACE [41]. Nevertheless, the predictive values of these biomarkers in patients with stable CAD and T2DM remains unclear.

To the best of our knowledge, this is the first study to evaluate whether the combined cardiac biomarkers approach incorporating hs-Tnl and BNP can provide incremental benefit in risk prediction of MACE in stable CAD patients with and without T2DM. In the present study, patients with T2DM were older and had a higher body mass index and prevalence of hypertension than those without T2DM. Furthermore, we observed a higher prevalence of elevated levels of hs-Tnl and BNP in patients with T2DM. Although the BNP concentration was significantly higher in T2DM patients, there was no significant difference in hs-Tnl level between patients with and without T2DM. Both elevated hs-Tnl and BNP were independently associated with the development of MACE among patients with and without T2DM. Interestingly, the predictive values of these cardiac biomarkers were stronger among CAD patients without T2DM. Our results revealed a more than twofold higher risk of MACE in patients without T2DM with elevated levels of hs-Tnl and BNP compared with patients with values below the cutoff level (Additional file 1: Table S3). Indeed, the prognostic value of these cardiac biomarkers was significantly higher in patients without T2DM than those with T2DM, after controlling for clinical risk factors.

### Multi-biomarker approach in CAD with or without T2DM

The multi-biomarker approach could be useful for stratification of high risk population for intensity risk factors control such as lipid lowering therapies [3, 4]. Prior studies [26] revealed that the combination of troponin and BNP provide the best prediction for cardiovascular events or death in patients with T2DM. Nevertheless, the predictive performance of these multi-biomarkers was not found to be better than the BNP alone for MACE. Indeed, we demonstrated that a combination of hs-TnI and BNP offered greater incremental value in risk prediction compared with the single biomarker models only in patients without T2DM. Furthermore, this multiple cardiac biomarker approach in patients without T2DM also provided significantly superior predictive power than in patients with T2DM, both for a single and combination of biomarkers approach. The overall effect of the proposed approach in T2DM patients follows the same trend as in those without T2DM but with a smaller magnitude as in previous studies [26]. The reasons for the lack of incremental predictive values of combined cardiac biomarkers in CAD patients with T2DM is unknown.

First, it is possible that similar mechanisms, such as microvascular injuries contribute to elevated hs-TnI and BNP in CAD and T2DM, and thus limited the incremental predictive values in using both biomarkers. In animal model of myocardial injury induced by carbon monoxide, there was a positive correlation between the levels of troponin and BNP [42]. Moreover, the levels of hs-Tnl and BNP are closely correlated with each other after myocardial infarction, and high levels of troponin may reflect the severity of heart failure [43]. Second, this may be due to factors such as renal impairment and hyperglycemia that contribute to both elevated hs-Tnl and BNP in patients with T2DM [44, 45]. Nevertheless, recent studies demonstrated that perioperative glycemic control with exenatide infusion does not affect troponin and BNP release after cardiac surgery [46]. Third, the presence of T2DM might induce different protective mechanisms, such as a more favorable lipoprotein prolife associated with elevated BNP via adiponectin signaling as cardioprotective effects [47]; and modifying myocardial response to ischemia/reperfusion mediated by dynamin-related protein 1 mitochondrial fission [48].

### Study limitations

This study has several limitations. First, there are no data that allow us to adjust for the severity of CAD, control of cardiovascular risk factors, such as blood pressure, smoking and lipid level, medications use and diabetes-related factors such as severity, glycemic controls and duration of diabetes that are known to influence the level of hs-Tnl and BNP [49, 50]. Second, we cannot conclude whether the biomarkers are causally related to the development of cardiovascular events. Third, in this study, other traditional risk factors, such as high-sensitive C-reactive protein and renal function were not included. Forth, it remains unclear whether repeated measurement of BNP and hs-TnI can provide further incremental value for prediction of MACE. Final, validation studies are needed to confirm our findings in CAD patients with and without T2DM.

## Conclusions

Elevated hs-Tnl and BNP levels are independent predictors of new-onset MACE in patients with stable CAD, the prognostic values of which are greater in patients without T2DM. Among patients without T2DM, risk factor model incorporating clinical risk factor and combination of biomarkers yielded the greatest predictive value. These results support the rationale of utilizing the multiple cardiac biomarkers approach as a prognostic tool for risk stratification in high-risk patients, and may contribute to the optimization of patient treatment and outcomes.

## Supplementary information

**Additional file 1: Table S1.** Unadjusted and age-adjusted Cox regression analysis predicting MACEs in stable CAD patients. **Table S2**. Competing risks regression models predicting MACEs in stable CAD patients. **Table S3.** Multivariable Cox regression models predicting MACEs in patients with and without T2DM.

## Data Availability

The datasets used and/or analyzed during the current study are available from the corresponding author on reasonable request.

## Abbreviations

ACS: acute coronary syndrome
AUC: area under the curve
BNP: B-type natriuretic peptide
CAD: coronary artery disease
CI: confidence interval
hs-Tnl: high-sensitivity troponin I
HR: hazard ratio
IDI: integrated discrimination improvement
MACE: major adverse cardiovascular event
NRI: net reclassification index
T2DM: type 2 diabetes mellitus
