# Supplementary material for: High-sensitivity troponin I and B-type natriuretic peptide biomarkers for prediction of cardiovascular events in patients with coronary artery disease with and without diabetes mellitus
Source: Cardiovasc Diabetol. 2019 Dec 17;18:171. doi: 10.1186/s12933-019-0974-2 (PMC6918569; doi:10.1186/s12933-019-0974-2)
Supplement: Supplementary file 1 — Additional file 1: Table S1. Unadjusted and age-adjusted Cox regression analysis predicting MACEs in stable CAD patients. Table S2. Competing risks regression models predicting MACEs in stable CAD patients. Table S3. Multivariable Cox regression models predicting MACEs in patients with and without T2DM. [file 12933_2019_974_MOESM1_ESM.docx]

| **Table S1. Unadjusted and age-adjusted Cox regression analysis predicting MACEs in stable CAD patients** | | | | |
| --- | --- | --- | --- | --- |
|  | Unadjusted | | Age-adjusted | |
| Characteristics | HR (95% CI) | *P* Value | HR (95% CI) | *P* Value |
| Age ≥65 years | 2.53 (2.00-3.21) | <0.001 | 2.53 (2.00-3.21) | <0.001 |
| Male | 0.71 (0.58-0.88) | 0.002 | 0.82 (0.67-1.01) | 0.067 |
| Current smoker | 0.90 (0.67-1.22) | 0.51 | 1.06 (0.78-1.43) | 0.72 |
| T2DM | 1.47 (1.13-1.90) | 0.004 | 1.39 (1.08-1.81) | 0.012 |
| Hypertension | 2.20 (1.43-3.39) | <0.001 | 1.80 (1.17-2.78) | 0.008 |
| BMI | 0.96 (0.94-0.99) | 0.014 | 0.98 (0.95-1.01) | 0.19 |
| Enrollment period* | 1.06 (0.93-1.21) | 0.39 | 1.08 (0.96-1.22) | 0.21 |
| BNP | 1.78 (1.46-2.16) | <0.001 | 1.68 (1.38-2.05) | <0.001 |
| hs-Tnl | 2.01 (1.64-2.47) | <0.001 | 1.95 (1.59-2.39) | <0.001 |
| *Log transformed before analysis. BNP and hs-Tnl are at levels above optimal cutoffs. Abbreviations: BMI, body mass index; BNP, brain natriuretic peptide; CI, confidence interval; HR, hazard ratio; hs-Tnl, high-sensitive troponin-I; T2DM, type 2 diabetes mellitus. | | | | |

| **Table S2. Competing risks regression models predicting MACEs in stable CAD patients** | | | | | | | | | | |
| --- | --- | --- | --- | --- | --- | --- | --- | --- | --- | --- |
|  | Model 1 | | Model 2 | | Model 3 | | Model 4 | | Model 5 | |
| Variable | SHR (95% CI) | *P* value | SHR (95% CI) | *P* value | SHR (95% CI) | *P* value | SHR (95% CI) | *P* value | SHR (95% CI) | *P* value |
| Age ≥65 years | 2.46  (1.94-3.11) | <0.001 | 2.29  (1.80-2.91) | <0.001 | 2.23  (1.75-2.83) | <0.001 | 2.24  (1.77-2.85) | <0.001 | 2.22  (1.74-2.82) | <0.001 |
| Male |  |  | 0.83  (0.68-1.03) | 0.089 | 0.84  (0.68-1.03) | 0.098 | 0.85  (0.69-1.05) | 0.14 | 0.85  (0.69-1.05) | 0.14 |
| T2DM | - | - | 1.29  (0.98-1.69) | 0.065 | 1.28  (0.98-1.68) | 0.072 | 1.35  (1.03-1.77) | 0.032 | 1.33  (1.01-1.75) | 0.040 |
| Hypertension | - | - | 1.54  (0.97-2.45) | 0.066 | 1.50  (0.94-2.38) | 0.088 | 1.52  (0.95-2.43) | 0.078 | 1.50  (0.94-2.39) | 0.092 |
| BNP | - | - | - | - | 1.63  (1.34-1.98) | <0.001 | - | - | 1.40  (1.14-1.71) | 0.001 |
| hs-Tnl | - | - | - | - | - | - | 1.93  (1.57-2.37) | <0.001 | 1.72  (1.39-2.14) | <0.001 |
| BNP and hs-Tnl are at levels above optimal cutoffs. Abbreviations: BNP, B-type natriuretic peptide; CAD, coronary artery disease; CI, confidence interval; SHR, subhazard ratio; hs-Tnl, high-sensitivity troponin I; MACE, major adverse cardiovascular events; T2DM, type 2 diabetes mellitus. | | | | | | | | | | |

| **Table S3. Multivariable Cox regression models predicting MACEs in patients with and without T2DM** | | | | |
| --- | --- | --- | --- | --- |
|  | T2DM | | No T2DM | |
| Characteristics | HR (95% CI) | *P* Value | HR (95% CI) | *P* Value |
| Age ≥65 years | 2.21 (1.70-2.88) | <0.001 | 2.45 (1.38-4.38) | 0.002 |
| Male | 0.84 (0.67-1.06) | 0.14 | 0.99 (0.60-1.66) | 0.98 |
| Hypertension | 1.24 (0.58-2.64) | 0.57 | 1.78 (1.00-3.19) | 0.051 |
| BNP | 1.29 (1.03-1.63) | 0.027 | 2.17 (1.32-3.57) | 0.002 |
| hs-Tnl | 1.69 (1.33-2.15) | <0.001 | 2.07 (1.27-3.35) | 0.003 |
| BNP and hs-Tnl are at levels above optimal cutoffs. Abbreviations: BNP, brain natriuretic peptide; CI, confidence interval; HR, hazard ratio; hs-Tnl, high-sensitive troponin-I; T2DM, type 2 diabetes mellitus. | | | | |
